# Critical and interactive digital health literacy and health information adoption among college students: a cross-sectional serial mediation study from a health communication perspective

**DOI:** 10.3389/fpubh.2026.1837883

**Published:** 2026-09-08

**Authors:** Yi Sun, Yang Zhao, Mingyu Zhao, Qian Ma

**Affiliations:** 1 College of Basic Medicine, Jining Medical University, Jining, China; 2 College of Foreign Languages, Weifang University, Weifang, Shandong, China; 3 School of Politics and Public Administration, Qufu Normal University, Rizhao, Shandong, China; 4 College of Pharmacy, Jining Medical University, Rizhao, China

**Keywords:** college students, cross-sectional study, digital health literacy, exercise self-efficacy, health information adoption, health risk perception

## Abstract

**Background:**

Digital media have transformed how college students access, evaluate, and use health information. Although digital health literacy has been linked with risk perception, preventive behavior, self-care, and information seeking, less is known about its association with health information adoption as a downstream stage of digital health communication and the statistical pathways that may account for this association.

**Objective:**

This study examined the cross-sectional association between critical and interactive digital health literacy and self-reported health information adoption among college students and estimated the specific indirect associations through health risk perception and exercise self-efficacy, separately and sequentially.

**Methods:**

This cross-sectional study used a structured online questionnaire administered in three batches to college students from 12 institutions in Shandong Province, China, between September and November 2025. An independent pilot sample (*n* = 331) informed item-level assessment and scale reduction, and 3,148 formal-survey responses were included in the primary analysis. The primary scores were a 10-item health information adoption score, a 9-item critical and interactive digital health literacy score, an 8-item health risk perception score, and a 10-item exercise self-efficacy score. Analyses included descriptive statistics, group comparisons, Pearson correlations, reliability and measurement-model assessment, and ordinary least-squares path models corresponding to PROCESS Model 6. The adjusted model controlled for gender, academic year, major category, residential background, family economic status, and weekly internet-use frequency. Percentile bootstrap confidence intervals were calculated from 5,000 resamples. Analyses were conducted using SPSS 26.0 and Python; additional models assessed survey-batch and alternative-scoring sensitivity.

**Results:**

Among the 3,148 participants, the mean scores were 30.83 (SD 8.24) for health information adoption, 29.21 (SD 6.25) for critical and interactive digital health literacy, 26.43 (SD 5.86) for health risk perception, and 32.35 (SD 8.17) for exercise self-efficacy. Digital health literacy was positively correlated with health information adoption (*r* = 0.416; *P* < 0.001). In the covariate-adjusted model, the total association between digital health literacy and health information adoption was *B* = 0.534. The direct association was *B* = 0.344 (64.3%). The specific indirect associations through health risk perception (effect = 0.090, bootstrap 95% CI 0.058–0.122; 16.9%), exercise self-efficacy (effect = 0.080, bootstrap 95% CI 0.059–0.101; 14.9%), and the serial health-risk-perception-to-exercise-self-efficacy pathway (effect = 0.020, bootstrap 95% CI 0.012–0.029; 3.8%) were statistically detectable. The total indirect association was 0.191 (bootstrap 95% CI 0.150–0.230; 35.7%). Results were materially similar after adjustment for survey batch and under alternative scoring specifications.

**Conclusions:**

Critical and interactive digital health literacy was positively associated with self-reported health information adoption among college students. Health risk perception and exercise self-efficacy provided separate and serial statistical indirect routes, but the direct association was the largest component and the serial route was comparatively small. Because the data were cross-sectional and self-reported, these estimates should be interpreted as statistical decompositions of associations rather than evidence of temporal or causal mediation.

## 1 Introduction

The pervasive integration of digital media into everyday life has fundamentally transformed how young populations access, interpret, evaluate, and utilize health information. In contrast to traditional communication contexts—where health knowledge was primarily acquired through formal education, family experience, and professional institutions—contemporary college students increasingly engage with health-related content via search engines, social media platforms, short-form video applications, online consultation services, and mobile health applications. Consequently, health communication is no longer a unidirectional process of information transmission but has evolved into a dynamic and iterative process encompassing information seeking, filtering, evaluation, interpretation, and application (1). Within this process, digital health literacy has emerged as a critical prerequisite enabling individuals to effectively participate in health communication and translate digital information resources into informed health decisions.

Classical scholarship defines eHealth literacy as the ability to seek, understand, appraise, and apply health information in digital environments to address health problems (2). More recent studies further emphasize that health literacy extends beyond individual cognitive competencies and constitutes an important social determinant shaping health opportunities and disparities (3). In contemporary digital environments, this competence is particularly important because individuals must not only locate online health information, but also judge its credibility, relevance, and applicability amid information overload, algorithmic recommendation, and uneven information quality.

In recent years, research on digital health literacy has expanded rapidly, with college students and young adults becoming a focal population. On the one hand, systematic reviews indicate that post-secondary students generally exhibit moderate to moderately high levels of eHealth literacy, with significant variability influenced by gender, socioeconomic status, digital experience, and information environments (4). On the other hand, eHealth literacy has been consistently associated with a range of cognitive, affective, and behavioral outcomes, particularly health knowledge, self-efficacy, preventive behaviors, information-seeking activities, self-care behaviors, and the utilization of online health services (4–6).

Importantly, the relationship between eHealth literacy and health-related outcomes has already been examined from multiple theoretical and empirical perspectives. For example, Jung and Song (7) demonstrated that risk perception mediated the association between eHealth literacy and infectious disease prevention behaviors among young adults based on the information–motivation–behavioral skills model. Soroya et al. (8) showed that Internet and social media exposure influenced self-care behavior through health anxiety, health literacy, and health information-seeking behavior. Similarly, Bin Naeem et al. (6) reported that eHealth literacy among undergraduate university students was positively associated with physical, psychological, and emotional self-care. These studies indicate that the broader association between eHealth literacy, risk cognition, information seeking, and health-related behavior has been substantially documented. Therefore, the present study does not claim that these general relationships remain unexplored.

Moreover, studies focusing on Generation *Z* highlight distinctive cohort characteristics in terms of information source preferences, platform selection, and trust formation, demonstrating that digital health literacy is closely related to health information seeking and health empowerment (9). Nevertheless, emerging evidence suggests that even individuals with relatively high levels of digital health literacy may still make suboptimal health decisions due to information overload, algorithm-driven exposure, or low-quality content (10). This indicates that digital health literacy does not necessarily translate directly into rational health behavior or into the acceptance of online health information as personally useful, credible, and actionable.

Against this background, the research gap addressed in the present study should be understood more precisely. Existing studies have mainly focused on preventive behaviors, self-care behaviors, online health information seeking, or general health-related decision-making. Comparatively less attention has been paid to health information adoption as a distinct downstream stage of digital health communication. Health information adoption differs from information exposure or information seeking because it concerns whether individuals accept, endorse, and incorporate online health information into their health-related judgments. In digital environments, exposure to health information does not necessarily lead to acceptance, and information seeking does not necessarily imply that the information obtained is trusted, internalized, or used. This distinction is particularly important among college students, who are highly exposed to digital health content but may vary considerably in their capacity and willingness to adopt such information in meaningful ways.

A further theoretical issue concerns how digital health literacy may be associated with health information adoption. Previous research has often explained health-related outcomes through a relatively linear ability–behavior framework, assuming that individuals with higher digital health literacy are more likely to make healthier decisions or engage in healthier behaviors. However, such a framework may overlook the intermediate cognitive and motivational processes through which digital competence becomes personally meaningful and practically actionable. Even individuals with relatively high digital health literacy may make suboptimal health decisions because of information overload, algorithm-driven exposure, inconsistent source credibility, or limited offline support for action (10). Thus, digital health literacy may be necessary but insufficient for explaining health information adoption.

In the present study, health information adoption is conceptually informed by the technology acceptance model (TAM). Following item reduction, the shortened measure retained indicators of perceived usefulness, perceived ease of use, attitudes toward internet use, and self-reported use of the internet to obtain health information. These indicators were organized into two closely related empirical factors: internet-use cognition and online health-information acquisition. Accordingly, health information adoption is operationalized here as students' favorable evaluation and self-reported uptake of online health information rather than as objectively observed behavior. TAM therefore provides the measurement basis for understanding health information adoption, but it does not fully explain why certain health information becomes personally relevant or why students may feel motivated to accept and use it in health-related judgment.

To address this limitation, health risk perception is introduced as a motivational appraisal component. Drawing on the logic of the Health Belief Model and health communication theory, health risk perception reflects individuals' subjective assessment of susceptibility to and severity of health threats. When students perceive a health issue as personally relevant and consequential, online health information may be more likely to be taken seriously and incorporated into decision-making. In this way, risk perception provides a theoretical bridge between digital information processing and motivational relevance. Nevertheless, the role of risk perception should not be assumed to be uniformly positive. While appropriate risk perception may increase attention to health information, excessive risk perception may induce anxiety, avoidance, or defensive processing (11). Its role in health information adoption is therefore likely to be context-dependent.

Exercise self-efficacy is further introduced as an action-oriented belief. Unlike general confidence in using digital technologies, exercise self-efficacy reflects students' perceived capability to maintain exercise under situational barriers. Its inclusion does not imply that all adopted health information concerns exercise; rather, it represents one domain-specific form of confidence in translating health-related knowledge into feasible health-promoting action. Drawing on social cognitive theory, exercise self-efficacy may therefore help explain why students who can evaluate online health information also differ in their confidence to act on health-related recommendations. Prior research has shown that self-efficacy may statistically account for part of the association between health literacy and online health information adoption or health-related behavioral intentions (10, 12). However, under conditions of uncertainty, limited resources, or low information trust, this association may weaken or be superseded by factors such as source credibility and platform trust (13).

The integration of digital health literacy, health risk perception, exercise self-efficacy, and health information adoption can also be understood through the information–motivation–behavioral skills logic. As operationalized in the revised analysis, digital health literacy primarily represents students' critical evaluation and interactive use of online health information; health risk perception reflects motivational appraisal and personal relevance; exercise self-efficacy represents exercise-related action confidence; and health information adoption represents a downstream evaluative and self-reported use outcome. Thus, the present model is not intended to mechanically combine TAM, the Health Belief Model, and social cognitive theory. Instead, these perspectives are used to distinguish information evaluation, risk appraisal, action confidence, and information adoption within the broader digital health communication process.

From a health communication perspective, the association between digital health literacy and health information adoption may extend beyond information access and evaluation to the way individuals construct meaning around health issues. First, risk perception may serve as a cognitive and motivational correlate linking digital competence with health information adoption. Information may be more likely to be taken seriously and incorporated into decision-making when individuals perceive it as personally relevant and consequential. Systematic reviews have reported positive associations between eHealth literacy and perceived susceptibility, perceived severity, and other risk-related constructs (4). Recent evidence also suggests that risk perception may statistically mediate the association between eHealth literacy and preventive behaviors among young adults (7). However, excessive risk perception may induce anxiety or information avoidance, thereby inhibiting information adoption (11), suggesting that its role is context-dependent.

Second, self-efficacy may function as a key behavioral mechanism facilitating the transition from “understanding” to “adoption.” Drawing on social cognitive theory, previous studies have shown that health literacy not only directly influences behavioral intentions but also is indirectly associated with health information adoption through enhanced self-efficacy in health management (12). Evidence from Chinese social media users indicates that self-efficacy significantly mediates the relationship between health literacy and online health information adoption (12). Similarly, research in online health communities has identified self-efficacy as a mediator between individual traits and health information adoption intentions (10). However, other studies suggest that under conditions of high uncertainty, the influence of self-efficacy may weaken or be supplanted by information trust (13), indicating that its role is not universally stable.

Importantly, risk perception and self-efficacy should not be viewed only as independent correlates; they may also constitute a potential sequential pathway linking digital health literacy with health information adoption. Students with stronger critical and interactive digital health literacy may be better able to recognize the personal relevance of health risks; when such appraisal is accompanied by confidence in action, they may also be more inclined to accept and use online health information. Existing research has reported associations among digital health literacy, self-efficacy, health attitudes, and behavioral outcomes (4, 14). Studies of information-source trust further indicate that individuals with lower health literacy may rely more heavily on non-professional sources, whereas those with higher literacy may place greater trust in authoritative sources (5). These findings support testing—without assuming—the proposed indirect associations.

However, the serial pathway from digital health literacy to risk perception, then to exercise self-efficacy, and finally to health information adoption should be treated cautiously (15–18). The pathway is theoretically plausible, but the cross-sectional design cannot establish temporal ordering or causal mediation. In addition, exercise self-efficacy is domain-specific and may capture only one possible route from risk appraisal to action confidence. Therefore, the serial indirect association is specified as exploratory and secondary rather than as the central explanatory mechanism.

**Figure 1:**
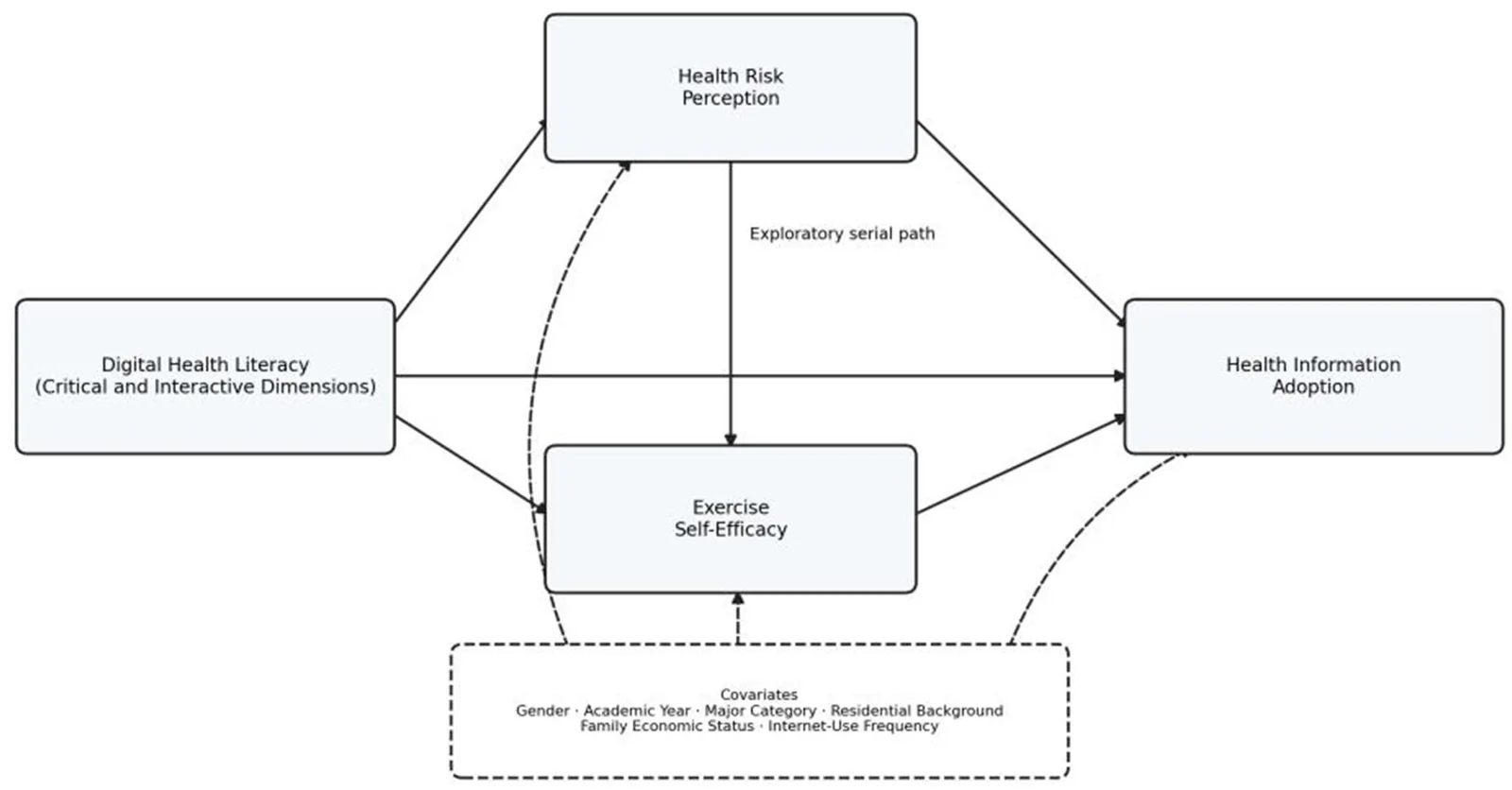
Revised conceptual model of direct and indirect associations. Solid arrows represent the hypothesized direct and indirect associations. Gender, academic year, major category, residential background, family economic status, and internet-use frequency were included as covariates in each endogenous-variable equation. Survey batch was included only in sensitivity analysis. The serial pathway through health risk perception and exercise self-efficacy was treated as exploratory and secondary.

Based on these considerations, this study positions critical and interactive digital health literacy within a health communication framework and focuses on health information adoption among college students. Health information adoption is treated as a downstream evaluative and self-reported use outcome within the broader information acquisition–evaluation–application continuum. Because the measure does not capture the full behavioral continuum, the study does not claim to assess active searching, systematic source comparison, information sharing, or sustained health behavior change. As shown in Figure 1, the proposed model tests direct and statistical indirect associations linking digital health literacy with health information adoption through health risk perception and exercise self-efficacy.

Based on the proposed model, the following hypotheses are formulated:

H1: Digital health literacy is positively associated with college students' health information adoption.

H2: Health risk perception statistically mediates the association between digital health literacy and health information adoption.

H3: Exercise self-efficacy statistically mediates the association between digital health literacy and health information adoption.

H4: As an exploratory hypothesis, health risk perception and exercise self-efficacy form a statistically detectable serial indirect association between digital health literacy and health information adoption.

This study contributes to the literature in three ways. First, it narrows attention from general online health information behavior to health information adoption, a more proximal evaluative and self-reported use outcome of digital health communication. Second, it examines health risk perception and exercise self-efficacy within a single explanatory model, linking risk appraisal with domain-specific action confidence while treating the serial pathway as exploratory. Third, it provides evidence from Chinese college students, a population situated within a distinctive higher-education and digital-media context. The findings may inform digital health education that strengthens critical information evaluation, calibrated risk recognition, and realistic action confidence.

Importantly, this study does not seek to overstate the novelty of the general association between digital health literacy and health-related outcomes. Its contribution lies instead in specifying health information adoption as a distinct downstream outcome and in testing whether risk appraisal and exercise-related action confidence provide additional statistical explanatory value beyond critical and interactive digital competence.

## 2 Methods

### 2.1 Participants

This cross-sectional study used a structured online questionnaire to recruit college students from 12 higher education institutions in Shandong Province, China, including junior colleges and undergraduate universities. Recruitment covered science, engineering, humanities, arts, and physical education, and sought coverage across academic years and major categories. Because participation was voluntary and invitations were distributed online, the procedure was a multi-institutional, quota-informed convenience recruitment strategy rather than proportionate stratified random sampling. Accordingly, the sample should not be interpreted as statistically representative of all college students in Shandong Province.

The formal survey was conducted in three batches between September and November 2025. The three batches were independent samples collected as part of the same cross-sectional study and used the same core measures, eligibility criteria, recruitment procedures, and response coding relevant to the present analysis. Trained student survey volunteers distributed the questionnaire link through class groups and student communication platforms. Participation was voluntary, and no financial or material incentives were provided. Before beginning the questionnaire, participants received information about the study purpose, anonymity, confidentiality, and their right to discontinue participation.

Before the three batch-specific datasets were merged, variable names, item identifiers, response coding, scoring direction, category labels, and the coding of the prespecified covariates were harmonized. The harmonized datasets were then appended by participant, and a survey-batch indicator was retained for sensitivity analysis. All eligible records across the three batches were included in the merged formal-survey dataset. A total of 3,148 analyzable questionnaires were included: 897 from batch 1, 836 from batch 2, and 1,415 from batch 3. The core scale items used in the primary analysis had no missing values. Because the available exports did not preserve a defensible denominator for all individuals who received or opened the survey link, a conventional response rate was not calculated. Possible self-selection and coverage bias should therefore be considered when interpreting the findings.

The questionnaire collected 11 demographic and internet-use characteristics. Descriptive analyses summarized the available characteristics, whereas the adjusted mediation models used six prespecified covariates that were consistently coded across all three batches: gender, academic year, major category, residential background, family economic status, and weekly internet-use frequency.

All respondents participated voluntarily and provided electronic informed consent before data collection. The study protocol was reviewed and approved by the Ethics Committee of Jining Medical University before participant recruitment and data collection (Approval No. JNMC-YX-2025-095). No directly identifying information was included in the analytic dataset.

### 2.2 Measures

#### 2.2.1 Health information adoption measure

Health information adoption was assessed using items adapted from the measurement framework proposed by Ahadzadeh et al. (15) and informed by the Technology Acceptance Model (TAM), which links adoption of online health information with perceived usefulness, ease of use, attitudes, and self-reported use.

The questionnaire administered 18 health-information-adoption items covering perceived usefulness, perceived ease, attitude, and self-reported use of the internet for health information. Item-level analysis of the independent pilot sample indicated substantial overlap among several items. To reduce redundancy while retaining conceptual coverage, the pre-specified primary outcome for the reanalysis was a 10-item reduced score comprising questionnaire items 58, 60, 61, 63, 64, 67, 68, 71, 72, and 75. The first six items represented evaluative cognition about internet use, and the final four represented perceived acquisition or use of online health information. Responses were coded from 1 (never) to 5 (always) and summed (range 10–50); higher scores indicated stronger self-reported health information adoption. The full 18-item score (range 18–90) was retained for sensitivity analysis.

Representative retained items included “Using the Internet helps me manage my daily health” and “I use the Internet to obtain health management information, such as exercise, smoking cessation, diet, nutrition, stress, and mental health.” Because the items measure evaluations and reported internet use, the resulting score was interpreted as self-reported information adoption rather than an objective measure of sustained health behavior change.

Psychometric evaluation used an independent pilot sample (*n* = 331) for item analysis and exploratory assessment, followed by confirmatory factor analysis in the merged formal sample (*n* = 3,148). The pilot sample was recruited separately from the formal-survey samples, and no pilot participant or record was included in any of the three formal-survey batches. Separating these stages reduced the risk of evaluating the exploratory solution in the same observations from which it was derived

In the pilot sample, the 18 administered items were suitable for factor analysis (KMO = 0.963; Bartlett's test: chi-square = 4,907.26, df = 153, *P* < 0.001). The first two eigenvalues were 11.10 and 1.18 and together accounted for 68.24% of variance. A two-component varimax solution was examined to preserve the two broad content domains; however, parallel analysis retained one dominant component. The reduced two-domain score should therefore be understood as a pragmatic content-preserving short form with a strong general factor, rather than as confirmation of four distinct TAM dimensions.

The retained items, their wording, domain assignment, item-total statistics, and the rationale for excluding the other eight items are reported in Supplementary Appendix 1: Item Composition and Scale Reduction (Supplementary Tables S1 – S3). Specifically, Supplementary Table S1 lists the administered and retained items and scoring rules, Supplementary Table S2 reports the item-level reliability diagnostics, and Supplementary Table S3 presents the pilot factor and parallel-analysis results. This reporting is intended to make the data-driven reduction transparent and reproducible.

In the formal sample, the 10-item score had high internal consistency (Cronbach alpha = 0.933), compared with alpha = 0.962 for the full 18-item score. The lower alpha after reduction, together with retained conceptual coverage, was considered preferable to relying on the highly redundant 18-item total. Item-level diagnostics are reported in Supplementary Appendix 1, Tables S1 – S3 and results using the full 18-item score are reported in Supplementary Appendix 3, Table S9.

In the formal sample, a correlated two-domain model for the 10 retained items showed CFI = 0.967, TLI = 0.956, RMSEA = 0.080, and SRMR = 0.033. Standardized loadings ranged from 0.649 to 0.836, composite reliability was 0.877–0.899, and AVE was 0.599–0.641. The latent-domain correlation was high (*r* = 0.908), consistent with a dominant general construct; therefore, the summed 10-item score was used in the primary analyses. These CFA estimates were obtained with maximum-likelihood estimation based on Pearson correlations and should be interpreted with the limitations of treating 5-category items as approximately continuous.

#### 2.2.2 Digital health literacy measure

Digital health literacy was measured using items from the scale developed by Jiang et al. (19). informed by Nutbeam's functional, interactive, and critical health-literacy framework (2, 3).

All 12 items were administered. Three negatively worded functional-literacy items concerning difficulty understanding formulas, text, and symbols (items 94–96) showed inconsistent direction and degraded reliability after conventional reverse scoring. The primary reanalysis therefore used the remaining 9 positively worded interactive and critical literacy items (items 89–93 and 97–100). Responses ranged from 1 (strongly disagree) to 5 (strongly agree) and were summed (range 9–45), with higher scores indicating stronger critical and interactive digital health literacy. This operationalization does not represent the complete three-level construct. The two alternative 12-item scoring rules and their reliability results are documented in Supplementary Appendix 2, Supplementary Table S6, and their mediation sensitivity results are reported in Supplementary Appendix 3, Supplementary Table S9.

The 9-item primary score had Cronbach alpha = 0.925. A correlated two-domain CFA showed CFI = 0.983, TLI = 0.976, RMSEA = 0.062, and SRMR = 0.023; standardized loadings ranged from 0.721 to 0.864, CR was 0.877–0.900, and AVE was 0.640–0.644. Because item removal was data-informed, the 9-item result is presented as the primary operational measure rather than as validation of a new standardized scale.

#### 2.2.3 Health risk perception measure

Health risk perception was measured using the 8-item Health Risk Perception Scale revised by Zhou Min. The measure covers perceived susceptibility and perceived severity and captures respondents' subjective appraisal of possible health damage to themselves or their family.

Four items assessed perceived susceptibility and four assessed perceived severity. Responses ranged from 1 (strongly disagree) to 5 (strongly agree) and were summed (range 8–40), with higher scores indicating stronger perceived health risk.

In the formal sample, the total score had Cronbach alpha = 0.908. The two domains had CR values of 0.809 and 0.898 and AVE values of 0.517 and 0.688, respectively.

The correlated two-factor CFA produced mixed fit (CFI = 0.931, TLI = 0.899, RMSEA = 0.132, SRMR = 0.063), and the factor correlation was high (*r* = 0.876). Thus, although reliability and residual fit were acceptable, the RMSEA and TLI did not support strong claims of structural validity. The total score was retained for comparability with the source measure, and this measurement limitation was considered when interpreting the mediation results.

#### 2.2.4 Exercise self-efficacy measure

Exercise self-efficacy was assessed using the 10-item Physical Exercise Self-Efficacy Scale developed by Jiang et al. (20) for college students. Grounded in Bandura's self-efficacy theory, the measure assesses confidence in maintaining exercise when facing situational and motivational barriers.

The items cover persistence despite lack of companionship, poor weather, environmental constraints, fatigue, boredom, schedule disruption, and limited visible effects. Responses ranged from 1 (very inconsistent with me) to 5 (very consistent with me) and were summed (range 10–50), with higher scores indicating stronger exercise-specific self-efficacy.

In the formal sample, Cronbach alpha was 0.944. A correlated two-domain CFA showed CFI = 0.976, TLI = 0.968, RMSEA = 0.072, and SRMR = 0.026. CR was 0.898–0.914 and AVE was 0.639–0.687. Because the two latent domains were highly correlated (*r* = 0.917), the total score was used in the mediation analysis.

#### 2.2.5 Covariates

The adjusted models included gender, academic year, major category, residential background, family economic status, and weekly internet-use frequency. These variables were selected *a priori* because they plausibly precede the focal exposure and may be associated with access to digital resources, health-information evaluation, risk appraisal, or exercise confidence. Categorical variables were indicator coded. Survey batch was not treated as a substantive confounder; it was added only in a sensitivity model to evaluate heterogeneity across the three data-collection batches. Because residual confounding remains possible in an observational cross-sectional study, covariate adjustment was not described as eliminating omitted-variable bias.

The primary operational definitions and psychometric results are summarized in Table 1. Detailed CFA fit indices, standardized loadings, CR, AVE, and factor correlations for all four measures are provided in Supplementary Appendix 2: Measurement-Model and Alternative-Scoring Results (Supplementary Tables S4 – S6).

**Table 1:** Psychometric properties of the primary study measures.

Construct	Items	Domains	Range	Alpha	CFA/validity evidence
Health information adoption	10	2 content domains; total score	10–50	0.933	CFI = 0.967; TLI = 0.956; RMSEA = 0.080; SRMR = 0.033; AVE = 0.599–0.641
Digital health literacy	9	2 domains (critical/interactive)	9–45	0.925	CFI = 0.983; TLI = 0.976; RMSEA = 0.062; SRMR = 0.023; AVE = 0.640–0.644
Health risk perception	8	2 domains; total score	8–40	0.908	CFI = 0.931; TLI = 0.899; RMSEA = 0.132; SRMR = 0.063; mixed structural fit
Exercise self-efficacy	10	2 domains; total score	10–50	0.944	CFI = 0.976; TLI = 0.968; RMSEA = 0.072; SRMR = 0.026; AVE = 0.639–0.687

### 2.3 Data analysis

Data management and statistical analyses were conducted using SPSS 26.0 and Python. All item codings and scale compositions were fixed consistently across the merged formal dataset. Harman's single-factor test was applied to the 37 items included in the primary composite scores. The first unrotated factor explained 34.68% of variance. This test was treated only as a limited diagnostic because it cannot rule out common-method bias in single-source, cross-sectional self-report data.

Internal consistency was assessed with Cronbach alpha. Pilot-sample item analysis of the 18 health-information-adoption items included the inter-item correlation matrix, corrected item-total correlations, alpha if item deleted, eigenvalues, varimax-rotated components, and parallel analysis. CFA was conducted in the formal sample using maximum-likelihood estimation of Pearson correlation matrices. Model evaluation emphasized CFI, TLI, RMSEA, and SRMR, together with standardized loadings, CR, AVE, and latent-factor correlations. Because the responses used 5-category ordinal scales, results from this continuous-indicator ML approach were interpreted cautiously; replication with an ordinal estimator is warranted.

Descriptive statistics included the mean, standard deviation, range, skewness, and kurtosis. Independent-samples *t* tests or one-way ANOVA examined demographic group differences. Variance homogeneity was assessed with Levene's test; Welch's test and Games–Howell comparisons were used when homogeneity was violated, otherwise conventional ANOVA and Tukey comparisons were used. Effect sizes were reported as Cohen d or eta squared. Pearson correlations summarized bivariate associations among the four primary composite scores.

Ordinary least-squares path models corresponding to Hayes PROCESS Model 6 were used to estimate the direct association, two specific single-mediator indirect associations, and the exploratory serial indirect association from digital health literacy through health risk perception and exercise self-efficacy to health information adoption. Percentile bootstrap 95% confidence intervals were calculated from 5,000 resamples using random seed 20260808; an indirect association was considered statistically detectable when its interval excluded zero. The primary adjusted model included the six covariates described above. An unadjusted model, a model additionally controlling for survey batch, a model using all 18 health-information-adoption items, and models using the two alternative 12-item digital-health-literacy scoring rules were conducted as sensitivity analyses. Full path coefficients are reported in Supplementary Appendix 3: Mediation and Sensitivity Analyses (Supplementary Table S7), bootstrap indirect associations in Supplementary Table S8, and the batch and alternative-scoring sensitivity models in Supplementary Table S9. Given the cross-sectional design and the absence of temporal ordering, all mediation estimates were interpreted as statistical indirect associations, not evidence of causal or longitudinal mediation.

## 3 Results

### 3.1 Common method bias

Harman's single-factor test of the 37 items included in the primary composite scores indicated that the first unrotated factor explained 34.68% of total variance. Although this value was below the frequently used 40% heuristic, the result does not demonstrate the absence of common-method bias. The single-source, cross-sectional self-report design therefore remains a limitation.

### 3.2 Descriptive statistics

Among the 3,148 participants, the mean health information adoption score was 30.83 (SD 8.24), the mean critical and interactive digital health literacy score was 29.21 (SD 6.25), the mean health risk perception score was 26.43 (SD 5.86), and the mean exercise self-efficacy score was 32.35 (SD 8.17) (Table 2). Because validated clinical or normative cut points are not available for these composite scores, the means were not categorized as low, moderate, or high.

**Table 2:** Descriptive statistics of the primary variables.

Variable	Items	Theoretical range	Mean	SD	Observed range	Skewness	Kurtosis
Health information adoption	10	10–50	30.83	8.24	10–50	0.055	0.362
Digital health literacy	9	9–45	29.21	6.25	9–45	−0.379	1.683
Health risk perception	8	8–40	26.43	5.86	8–40	−0.541	1.402
Exercise self-efficacy	10	10–50	32.35	8.17	10–50	−0.393	0.542

*N* = 3,148. Digital health literacy refers to the 9-item critical and interactive score used in the primary analysis.

### 3.3 Differences across demographic characteristics

Female students scored higher than male students on health information adoption, digital health literacy, and health risk perception, whereas male students scored slightly higher on exercise self-efficacy; all four gender differences were statistically detectable but small (absolute Cohen *d* = 0.102–0.261; Table 3). Across the other demographic characteristics, major-category differences were detected for all four outcomes. Health information adoption also differed by residential background and family economic status, whereas academic-year differences were not detected for any outcome (Table 4). The full group-specific means, robust tests, and *post-hoc* comparisons are provided in Supplementary Appendix 4, Tables S10–S11. Batch differences were small and are reported in Supplementary Table S12. These exploratory group comparisons were not used as a significance-based procedure for selecting covariates.

**Table 3:** Gender differences in the primary variables.

Variable	Gender	*n*	Mean	SD	Test	Statistic	*P*	Cohen *d*
Health information adoption	Male	1,520	30.09	8.71	Welch *t*	4.858	<0.001	0.174
	Female	1,628	31.52	7.71				
Digital health literacy	Male	1,520	28.38	6.60	Student *t*	7.312	<0.001	0.261
	Female	1,628	30.00	5.80				
Health risk perception	Male	1,520	25.66	6.21	Student *t*	7.109	<0.001	0.254
	Female	1,628	27.14	5.42				
Exercise self-efficacy	Male	1,520	32.78	8.32	Student *t*	−2.869	0.004	−0.102
	Female	1,628	31.95	8.01				

Positive Cohen d values indicate higher scores among female students because the contrast was female minus male. The exercise self-efficacy contrast was negative.

**Table 4:** Omnibus demographic group comparisons.

Outcome	Characteristic	Method	*F*	df	*P*	Eta squared
Health information adoption	Academic year	One-way ANOVA	1.498	3,3144	0.213	0.001
Health information adoption	Major category	One-way ANOVA	6.867	3,3144	<0.001	0.007
Health information adoption	Residential background	One-way ANOVA	10.454	2,3145	<0.001	0.007
Health information adoption	Family economic status	Welch ANOVA	12.618	2,125.0	<0.001	0.008
Digital health literacy	Academic year	One-way ANOVA	2.242	3,3144	0.081	0.002
Digital health literacy	Major category	Welch ANOVA	13.045	3,1499.4	<0.001	0.012
Digital health literacy	Residential background	One-way ANOVA	2.538	2,3145	0.079	0.002
Digital health literacy	Family economic status	Welch ANOVA	12.092	2,124.7	<0.001	0.008
Health risk perception	Academic year	One-way ANOVA	0.315	3,3144	0.814	0.000
Health risk perception	Major category	One-way ANOVA	9.406	3,3144	<0.001	0.009
Health risk perception	Residential background	Welch ANOVA	2.823	2,1523.9	0.060	0.002
Health risk perception	Family economic status	One-way ANOVA	2.695	2,3145	0.068	0.002
Exercise self-efficacy	Academic year	One-way ANOVA	1.308	3,3144	0.270	0.001
Exercise self-efficacy	Major category	Welch ANOVA	6.431	3,1477.2	<0.001	0.007
Exercise self-efficacy	Residential background	Welch ANOVA	0.518	2,1536.3	0.596	0.000
Exercise self-efficacy	Family economic status	One-way ANOVA	1.548	2,3145	0.213	0.001

Welch ANOVA was used when the homogeneity assumption was violated. Complete group means and *post-hoc* comparisons are reported in Supplementary Tables S10 and S11.

### 3.4 Correlation analysis

All four primary composite scores were positively correlated (all *P* < 0.001; Table 5). Digital health literacy correlated with health information adoption (*r* = 0.416), health risk perception (*r* = 0.560), and exercise self-efficacy (*r* = 0.414). Health risk perception correlated with health information adoption (*r* = 0.340) and exercise self-efficacy (*r* = 0.333), while exercise self-efficacy correlated with health information adoption (*r* = 0.322). These cross-sectional correlations describe co-occurrence and do not establish temporal or causal ordering.

**Table 5:** Pearson correlations among the primary variables.

Variable	1	2	3	4
1. Health information adoption	—			
2. Digital health literacy	0.416^***^	—		
3. Health risk perception	0.340^***^	0.560^***^	—	
4. Exercise self-efficacy	0.322^***^	0.414^***^	0.333^***^	—

*N* = 3,148. ^***^P < 0.001, two-tailed.

### 3.5 Mediation analysis

In the covariate-adjusted path model, digital health literacy was positively associated with health risk perception (*B* = 0.511, SE = 0.014, *P* < 0.001) and exercise self-efficacy after adjustment for health risk perception (*B* = 0.453, SE = 0.025, *P* < 0.001). Health risk perception was positively associated with exercise self-efficacy (*B* = 0.227, SE = 0.027, *P* < 0.001). In the health information adoption equation, digital health literacy (*B* = 0.344, SE = 0.027), health risk perception (*B* = 0.177, SE = 0.027), and exercise self-efficacy (*B* = 0.176, SE = 0.018) were each positively associated with the outcome (all *P* < 0.001; Table 6). The respective equation R-squared values were 0.327, 0.212, and 0.221.

**Table 6:** Focal path coefficients in the covariate-adjusted serial mediation model.

Path	*B*	SE	*t*	*P*	95% CI lower	95% CI upper
DHL → HRP	0.511	0.014	36.498	<0.001	0.484	0.538
DHL → ESE	0.453	0.025	17.985	<0.001	0.404	0.503
HRP → ESE	0.227	0.027	8.417	<0.001	0.174	0.280
DHL → HIA (direct)	0.344	0.027	12.947	<0.001	0.292	0.396
HRP → HIA	0.177	0.027	6.467	<0.001	0.123	0.230
ESE → HIA	0.176	0.018	9.826	<0.001	0.141	0.211

*N* = 3,148. All coefficients are unstandardized. Each endogenous-variable equation adjusted for gender, academic year, major category, residential background, family economic status, and weekly internet-use frequency. DHL, digital health literacy; HRP, health risk perception; ESE, exercise self-efficacy; HIA, health information adoption.

The total association between digital health literacy and health information adoption was *B* = 0.534, of which the direct association was *B* = 0.344 (64.3%). The total indirect association was 0.191 (35.7%; bootstrap 95% CI 0.150–0.230). The specific indirect associations through health risk perception (0.090; 16.9%), exercise self-efficacy (0.080; 14.9%), and the serial health-risk-perception-to-exercise-self-efficacy pathway (0.020; 3.8%) all had bootstrap intervals excluding zero (Table 7). The serial association was smaller than either single-mediator association and was treated as exploratory. These estimates are statistical decompositions of cross-sectional associations rather than causal mediation effects. Results were materially similar after additional adjustment for survey batch and under alternative scoring specifications (Supplementary Appendix 3, Tables S7–S9).

**Table 7:** Direct and indirect associations in the covariate-adjusted model.

Association	Effect	SE	95% CI lower	95% CI upper	Proportion, %
Total association	0.534	0.022	0.492	0.577	100.0
Direct association	0.344	0.027	0.292	0.396	64.3
Total indirect association	0.191	0.020	0.150	0.230	35.7
DHL → HRP → HIA	0.090	0.016	0.058	0.122	16.9
DHL → ESE → HIA	0.080	0.011	0.059	0.101	14.9
DHL → HRP → ESE → HIA	0.020	0.004	0.012	0.029	3.8

Indirect-association SEs and confidence intervals are based on 5,000 percentile bootstrap resamples (seed = 20260808). Proportions equal effect divided by the total association. The total and direct confidence intervals are ordinary least-squares intervals; the indirect intervals are bootstrap intervals.

## 4 Discussion

From a health communication perspective, this study examined cross-sectional associations among critical and interactive digital health literacy, health risk perception, exercise self-efficacy, and self-reported health information adoption among college students. All four composite scores were positively correlated. In the adjusted model, digital health literacy was associated directly with health information adoption and indirectly through health risk perception and exercise self-efficacy. The direct association accounted for 64.3% of the total association, whereas the three specific indirect associations accounted for 16.9%, 14.9%, and 3.8%, respectively. Thus, the two single-mediator routes were more prominent than the exploratory serial route. These findings describe partial statistical pathways and should not be interpreted as evidence of temporal or causal mediation.

The results are consistent with a conversion gap between the ability to evaluate digital health information and its reported adoption. Digital health literacy was positively related to adoption, but the outcome did not capture the full progression from exposure and searching to source comparison, sharing, and sustained behavior change. Accordingly, higher literacy should not be assumed to translate automatically into objective action. Nutbeam and Lloyd (3) emphasized that health literacy affects opportunities to act as well as comprehension, and previous reviews have linked eHealth literacy with cognitive, emotional, and behavioral outcomes among postsecondary students (4). The present study adds a narrower adoption-oriented outcome, while recognizing that platform design, information overload, trust, usefulness, and personal relevance may shape whether information is accepted or used (1, 10).

The demographic findings also require a more cautious interpretation than the original analysis suggested. Female students had higher mean scores for health information adoption, digital health literacy, and health risk perception, whereas male students had slightly higher exercise self-efficacy. All four gender differences were statistically detectable, but their effect sizes were small. Major-category differences were detected for all four outcomes; health information adoption additionally differed by residential background and family economic status. Academic-year differences were not detected. Because the study used voluntary online recruitment and uneven subgroup sizes, these comparisons should be treated as descriptive rather than as population estimates. They nevertheless suggest that digital health communication may reflect differences in social resources and educational context as well as individual competence.

The strongest bivariate association was between digital health literacy and health risk perception (*r* = 0.560), followed by the association between digital health literacy and health information adoption (*r* = 0.416). This pattern is compatible with the proposition that critical evaluation of online information is related to recognizing and interpreting health threats. However, correlations cannot establish direction, and risk perception may also motivate information evaluation. Digital health literacy should therefore be understood as an enabling correlate rather than a sufficient condition for adoption; source credibility, platform trust, perceived usefulness, and perceived relevance may also influence the outcome (13).

Digital health literacy retained a direct association with health information adoption after adjustment for the six demographic and internet-use covariates and both proposed mediators (*B* = 0.344). The predominance of this direct component indicates that the model was not fully mediated. More proximal evaluative processes, including perceived usefulness, ease of use, information quality, and habitual internet use, may account for part of the remaining association. Sensitivity analyses using the full 18-item adoption score, alternative 12-item digital-health-literacy scoring rules, and additional batch adjustment did not change the general pattern, although the magnitude varied across scoring specifications.

Health risk perception provided one statistical indirect route (effect = 0.090, bootstrap 95% CI 0.058–0.122). Students with higher digital health literacy also tended to report greater perceived susceptibility and severity, which in turn was associated with greater adoption. This pattern is compatible with health-belief reasoning because perceived risk can increase the personal relevance of health information. Nevertheless, the health-risk-perception measurement model showed mixed fit, including RMSEA = 0.132 and TLI = 0.899, and its factor correlation was high. The indirect association should therefore be interpreted cautiously rather than as definitive confirmation of a distinct theoretical mechanism.

Exercise self-efficacy provided a second statistical indirect route (effect = 0.080, bootstrap 95% CI 0.059–0.101). This suggests that students who reported stronger digital health literacy also tended to report greater confidence in maintaining exercise under barriers, which was associated with higher information adoption. The result is consistent with evidence linking health literacy, self-efficacy, and behavioral intentions (12). At the same time, exercise self-efficacy is domain-specific; it should not be treated as a general measure of the ability to implement every type of online health advice. Time, resources, peer support, and campus conditions may affect whether confidence can be translated into action.

The exploratory serial indirect association through health risk perception and then exercise self-efficacy was statistically detectable (effect = 0.020, bootstrap 95% CI 0.012–0.029) but represented only 3.8% of the total association. It was smaller than either single-mediator route and should remain secondary in the interpretation. Moreover, the cross-sectional data do not demonstrate that literacy preceded risk perception or that risk perception preceded self-efficacy. Longitudinal or experimental studies are needed to test temporal ordering and to compare this sequence with alternatives involving trust, credibility, social support, platform experience, or campus health environments.

Theoretically, the study narrows attention from broad online health-information behavior to a downstream evaluative and self-reported use outcome. The findings support an integrated but limited account in which critical and interactive literacy, risk appraisal, and exercise-related confidence are associated with adoption. Practically, universities may combine source-evaluation training with calibrated risk communication, realistic action planning, and supportive offline opportunities. However, the findings do not justify assuming that increasing risk perception alone will improve behavior, nor do they establish that the proposed interventions would cause greater adoption. Programs should be evaluated prospectively and should consider students with fewer economic or residential resources without stigmatizing those groups.

Several limitations should be acknowledged. First, the cross-sectional design precludes temporal and causal conclusions. Second, voluntary online recruitment from institutions in one province, the absence of a defensible invitation denominator, and uneven subgroup sizes limit representativeness and prevent calculation of a conventional response rate. Third, all focal variables were self-reported; Harman's test cannot exclude common-method, recall, or social-desirability bias. Fourth, the outcome was a data-informed 10-item short score and the primary predictor was a data-informed 9-item critical and interactive score; although full-scale and alternative-scoring sensitivity analyses were conducted, these operationalizations require independent replication. Fifth, parallel analysis indicated one dominant adoption component, the two adoption domains were highly correlated, and the health-risk-perception CFA showed mixed fit. Sixth, CFA used maximum likelihood with Pearson correlations for 5-category items; ordinal estimators should be used in future validation. Finally, the outcome did not assess objective searching, source comparison, sharing, or sustained behavior change. Multi-site longitudinal studies using objective indicators and preregistered measurement models are needed.

## 5 Conclusion

Critical and interactive digital health literacy was positively associated with self-reported health information adoption among college students. Health risk perception and exercise self-efficacy each provided statistical indirect routes, while the exploratory serial route was smaller and accounted for 3.8% of the total association. Most of the association remained direct. These cross-sectional findings support a cautious, multifactor interpretation involving information evaluation, risk appraisal, and exercise-related confidence, but they do not establish causal mediation. Longitudinal and experimental research with independently validated measures is required.

## Data Availability

The raw data supporting the conclusions of this article will be made available by the authors, without undue reservation.
