# Supplementary material for: Critical and interactive digital health literacy and health information adoption among college students: a cross-sectional serial mediation study from a health communication perspective
Source: Front Public Health. 2026 Sep 8;14:1837883. doi: 10.3389/fpubh.2026.1837883 (PMC13593750; doi:10.3389/fpubh.2026.1837883)
Supplement: Supplementary file 1 [file Table_1.docx]

**Supplementary Materials**

**Supplementary Appendix 1. Item Composition and Scale Reduction**

| Construct | Item | Item wording (English translation) | Primary score | Domain | Response coding |
| --- | --- | --- | --- | --- | --- |
| HIA | 58 | Using the internet helps me manage my daily health. | Yes | Evaluative cognition | Never=1 to Always=5 |
| HIA | 59 | Using the internet to obtain health information helps me manage my health better. | No | Evaluative cognition | Never=1 to Always=5 |
| HIA | 60 | Using the internet to obtain health information is beneficial to me. | Yes | Evaluative cognition | Never=1 to Always=5 |
| HIA | 61 | It is convenient to search online for health information and learn health-management skills. | Yes | Evaluative cognition | Never=1 to Always=5 |
| HIA | 62 | Using the internet reduces the time needed to obtain health-related information and manage my health. | No | Evaluative cognition | Never=1 to Always=5 |
| HIA | 63 | Searching for health information and managing health through the internet require less effort than using other media. | Yes | Evaluative cognition | Never=1 to Always=5 |
| HIA | 64 | Using the internet for health information and health management is a good approach. | Yes | Evaluative cognition | Never=1 to Always=5 |
| HIA | 65 | Using the internet for health information and health management would be a wise idea. | No | Evaluative cognition | Never=1 to Always=5 |
| HIA | 66 | I like using the internet for health information and health management. | No | Evaluative cognition | Never=1 to Always=5 |
| HIA | 67 | Using the internet for health information and health management would be an enjoyable experience. | Yes | Evaluative cognition | Never=1 to Always=5 |
| HIA | 68 | I use the internet to obtain information about medications. | Yes | Acquisition/use | Never=1 to Always=5 |
| HIA | 69 | I use the internet to obtain information before or after a physician makes a decision. | No | Acquisition/use | Never=1 to Always=5 |
| HIA | 70 | I use the internet to obtain descriptions of various diseases. | No | Acquisition/use | Never=1 to Always=5 |
| HIA | 71 | I use the internet to obtain information about treatments, therapies, or diagnoses. | Yes | Acquisition/use | Never=1 to Always=5 |
| HIA | 72 | I use the internet to obtain information about how to care for myself. | Yes | Acquisition/use | Never=1 to Always=5 |
| HIA | 73 | I use the internet to decide how to treat an illness. | No | Acquisition/use | Never=1 to Always=5 |
| HIA | 74 | I use the internet to learn how to cope with an illness. | No | Acquisition/use | Never=1 to Always=5 |
| HIA | 75 | I use the internet to obtain health-management information (eg, exercise, alcohol cessation, smoking cessation, diet, nutrition, stress, and mental health). | Yes | Acquisition/use | Never=1 to Always=5 |
| DHL | 89 | When I have doubts about online health information, I verify it through other channels. | Yes | Critical/interactive | Strongly disagree=1 to Strongly agree=5 |
| DHL | 90 | I review the opinions of many people in comment sections before making a health-related decision or taking action. | Yes | Critical/interactive | Strongly disagree=1 to Strongly agree=5 |
| DHL | 91 | I examine the validity and reliability of online health information. | Yes | Critical/interactive | Strongly disagree=1 to Strongly agree=5 |
| DHL | 92 | I try to verify the accuracy of online health information using different sources. | Yes | Critical/interactive | Strongly disagree=1 to Strongly agree=5 |
| DHL | 93 | I consider whether online health information applies to me. | Yes | Critical/interactive | Strongly disagree=1 to Strongly agree=5 |
| DHL | 94 | I find mathematical formulas in online health information (eg, algorithms for calories burned) difficult to calculate. | No | Functional difficulty | Strongly disagree=1 to Strongly agree=5 |
| DHL | 95 | I find the written content of online health information difficult to understand. | No | Functional difficulty | Strongly disagree=1 to Strongly agree=5 |
| DHL | 96 | I cannot understand the meaning of symbols used in online health information (eg, body mass index [BMI]). | No | Functional difficulty | Strongly disagree=1 to Strongly agree=5 |
| DHL | 97 | I can understand the online health information I obtain. | Yes | Critical/interactive | Strongly disagree=1 to Strongly agree=5 |
| DHL | 98 | I can select the information I need from online health information. | Yes | Critical/interactive | Strongly disagree=1 to Strongly agree=5 |
| DHL | 99 | I pay attention to acquiring new knowledge from online health information. | Yes | Critical/interactive | Strongly disagree=1 to Strongly agree=5 |
| DHL | 100 | I can efficiently find online health information through search engines (eg, Baidu or Google). | Yes | Critical/interactive | Strongly disagree=1 to Strongly agree=5 |
| HRP | 102 | I feel that my health or my family members’ health may currently be at risk. | Yes | Susceptibility | Strongly disagree=1 to Strongly agree=5 |
| HRP | 103 | I feel that my health or my family members’ health may be at risk in the future. | Yes | Susceptibility | Strongly disagree=1 to Strongly agree=5 |
| HRP | 104 | I believe that physical condition has a substantial influence on health impairment. | Yes | Susceptibility | Strongly disagree=1 to Strongly agree=5 |
| HRP | 105 | I am very worried that my health or my family members’ health may be impaired. | Yes | Susceptibility | Strongly disagree=1 to Strongly agree=5 |
| HRP | 106 | Health impairment would adversely affect my social life. | Yes | Severity | Strongly disagree=1 to Strongly agree=5 |
| HRP | 107 | The consequences of health impairment would be very serious. | Yes | Severity | Strongly disagree=1 to Strongly agree=5 |
| HRP | 108 | Health impairment would increase my expenses. | Yes | Severity | Strongly disagree=1 to Strongly agree=5 |
| HRP | 109 | Health impairment would cause physical discomfort. | Yes | Severity | Strongly disagree=1 to Strongly agree=5 |
| ESE | 110 | Even without anyone accompanying me, I would continue with my exercise plan. | Yes | Situational persistence | Very inconsistent=1 to Very consistent=5 |
| ESE | 111 | Even when the weather is very bad, I would continue exercising. | Yes | Situational persistence | Very inconsistent=1 to Very consistent=5 |
| ESE | 112 | Even when I could watch television or play video games, I would still exercise in my free time. | Yes | Situational persistence | Very inconsistent=1 to Very consistent=5 |
| ESE | 113 | Even after moving to a different environment, I would find a way to continue exercising. | Yes | Situational persistence | Very inconsistent=1 to Very consistent=5 |
| ESE | 114 | Even when exercise facilities or conditions are limited, I can find a way to exercise. | Yes | Situational persistence | Very inconsistent=1 to Very consistent=5 |
| ESE | 115 | Even when other matters delay my exercise, I would find a way to make up the planned exercise. | Yes | Situational persistence | Very inconsistent=1 to Very consistent=5 |
| ESE | 116 | Even when I feel tired during exercise, I can persist until I complete it. | Yes | Motivational persistence | Very inconsistent=1 to Very consistent=5 |
| ESE | 117 | If I become bored after exercising for a period, I would make active adjustments and continue exercising. | Yes | Motivational persistence | Very inconsistent=1 to Very consistent=5 |
| ESE | 118 | Even when exercise is difficult, I am confident that I can complete it. | Yes | Motivational persistence | Very inconsistent=1 to Very consistent=5 |
| ESE | 119 | Even when I do not notice clear exercise effects, I would continue exercising. | Yes | Motivational persistence | Very inconsistent=1 to Very consistent=5 |

**Table S1. Administered and retained questionnaire items, response coding, and domain assignments**

*Note.* HIA10 retained Q58, Q60, Q61, Q63, Q64, Q67, Q68, Q71, Q72, and Q75. DHL9 retained Q89–Q93 and Q97–Q100. The corrected Q69 wording is shown; Q69 was not included in HIA10.

**Table S2A. Inter-item correlation matrix for the 18 administered Health Information Adoption items**

| Item | 58 | 59 | 60 | 61 | 62 | 63 | 64 | 65 | 66 | 67 | 68 | 69 | 70 | 71 | 72 | 73 | 74 | 75 |
| --- | --- | --- | --- | --- | --- | --- | --- | --- | --- | --- | --- | --- | --- | --- | --- | --- | --- | --- |
| 58 | — |  |  |  |  |  |  |  |  |  |  |  |  |  |  |  |  |  |
| 59 | 0.66 | — |  |  |  |  |  |  |  |  |  |  |  |  |  |  |  |  |
| 60 | 0.56 | 0.65 | — |  |  |  |  |  |  |  |  |  |  |  |  |  |  |  |
| 61 | 0.54 | 0.61 | 0.68 | — |  |  |  |  |  |  |  |  |  |  |  |  |  |  |
| 62 | 0.54 | 0.58 | 0.60 | 0.66 | — |  |  |  |  |  |  |  |  |  |  |  |  |  |
| 63 | 0.51 | 0.55 | 0.58 | 0.64 | 0.66 | — |  |  |  |  |  |  |  |  |  |  |  |  |
| 64 | 0.52 | 0.56 | 0.61 | 0.67 | 0.65 | 0.71 | — |  |  |  |  |  |  |  |  |  |  |  |
| 65 | 0.53 | 0.57 | 0.60 | 0.62 | 0.62 | 0.68 | 0.71 | — |  |  |  |  |  |  |  |  |  |  |
| 66 | 0.50 | 0.55 | 0.60 | 0.60 | 0.60 | 0.66 | 0.70 | 0.68 | — |  |  |  |  |  |  |  |  |  |
| 67 | 0.48 | 0.55 | 0.57 | 0.58 | 0.60 | 0.63 | 0.66 | 0.69 | 0.71 | — |  |  |  |  |  |  |  |  |
| 68 | 0.44 | 0.49 | 0.55 | 0.56 | 0.57 | 0.60 | 0.62 | 0.59 | 0.63 | 0.67 | — |  |  |  |  |  |  |  |
| 69 | 0.42 | 0.48 | 0.53 | 0.50 | 0.54 | 0.52 | 0.56 | 0.58 | 0.61 | 0.61 | 0.62 | — |  |  |  |  |  |  |
| 70 | 0.44 | 0.51 | 0.58 | 0.59 | 0.58 | 0.59 | 0.63 | 0.64 | 0.64 | 0.65 | 0.65 | 0.64 | — |  |  |  |  |  |
| 71 | 0.45 | 0.50 | 0.54 | 0.53 | 0.55 | 0.57 | 0.58 | 0.61 | 0.61 | 0.63 | 0.63 | 0.63 | 0.70 | — |  |  |  |  |
| 72 | 0.49 | 0.52 | 0.55 | 0.56 | 0.56 | 0.57 | 0.61 | 0.64 | 0.61 | 0.60 | 0.62 | 0.62 | 0.66 | 0.70 | — |  |  |  |
| 73 | 0.43 | 0.46 | 0.50 | 0.47 | 0.49 | 0.47 | 0.52 | 0.55 | 0.55 | 0.56 | 0.54 | 0.56 | 0.60 | 0.63 | 0.65 | — |  |  |
| 74 | 0.45 | 0.49 | 0.53 | 0.53 | 0.53 | 0.53 | 0.56 | 0.57 | 0.58 | 0.58 | 0.59 | 0.56 | 0.61 | 0.64 | 0.67 | 0.67 | — |  |
| 75 | 0.47 | 0.53 | 0.55 | 0.56 | 0.55 | 0.57 | 0.59 | 0.62 | 0.61 | 0.62 | 0.59 | 0.57 | 0.62 | 0.63 | 0.66 | 0.61 | 0.70 | — |

*Note.* Lower-triangular Pearson correlations are shown; all items were coded 1–5 in the same direction.

**Table S2B. Pilot item-level reliability diagnostics for the 18 Health Information Adoption items**

| Item | Retained | Mean | SD | Corrected item–total r | Alpha if deleted | Mean inter-item r |
| --- | --- | --- | --- | --- | --- | --- |
| 58 | Yes | 2.994 | 1.042 | 0.693 | 0.962 | 0.547 |
| 59 | No | 3.042 | 1.020 | 0.723 | 0.962 | 0.569 |
| 60 | Yes | 3.187 | 1.001 | 0.741 | 0.961 | 0.582 |
| 61 | Yes | 3.311 | 1.010 | 0.729 | 0.961 | 0.573 |
| 62 | No | 3.193 | 0.999 | 0.743 | 0.961 | 0.584 |
| 63 | Yes | 3.284 | 1.017 | 0.775 | 0.961 | 0.609 |
| 64 | Yes | 3.251 | 1.012 | 0.757 | 0.961 | 0.595 |
| 65 | No | 3.257 | 1.005 | 0.757 | 0.961 | 0.595 |
| 66 | No | 3.281 | 0.974 | 0.772 | 0.961 | 0.606 |
| 67 | Yes | 3.269 | 0.968 | 0.819 | 0.960 | 0.641 |
| 68 | Yes | 3.254 | 0.989 | 0.785 | 0.961 | 0.616 |
| 69 | No | 3.100 | 1.044 | 0.735 | 0.961 | 0.578 |
| 70 | No | 3.242 | 0.983 | 0.811 | 0.960 | 0.635 |
| 71 | Yes | 3.166 | 0.991 | 0.737 | 0.961 | 0.579 |
| 72 | Yes | 3.233 | 1.011 | 0.750 | 0.961 | 0.590 |
| 73 | No | 3.076 | 1.046 | 0.739 | 0.961 | 0.581 |
| 74 | No | 3.157 | 1.009 | 0.761 | 0.961 | 0.597 |
| 75 | Yes | 3.215 | 1.012 | 0.768 | 0.961 | 0.603 |

*Note.* Pilot n=331; Cronbach alpha for all 18 items=0.963.

**Table S3A. Pilot two-component loadings for the 18 Health Information Adoption items**

| Item | Retained | Component 1 | Component 2 | Communality |
| --- | --- | --- | --- | --- |
| 58 | Yes | 0.306 | 0.728 | 0.623 |
| 59 | No | 0.348 | 0.724 | 0.645 |
| 60 | Yes | 0.271 | 0.829 | 0.761 |
| 61 | Yes | 0.354 | 0.727 | 0.653 |
| 62 | No | 0.451 | 0.646 | 0.622 |
| 63 | Yes | 0.429 | 0.713 | 0.693 |
| 64 | Yes | 0.382 | 0.738 | 0.691 |
| 65 | No | 0.441 | 0.678 | 0.654 |
| 66 | No | 0.542 | 0.592 | 0.644 |
| 67 | Yes | 0.622 | 0.570 | 0.713 |
| 68 | Yes | 0.716 | 0.429 | 0.697 |
| 69 | No | 0.739 | 0.338 | 0.660 |
| 70 | No | 0.703 | 0.476 | 0.721 |
| 71 | Yes | 0.804 | 0.273 | 0.720 |
| 72 | Yes | 0.770 | 0.326 | 0.700 |
| 73 | No | 0.786 | 0.293 | 0.704 |
| 74 | No | 0.770 | 0.338 | 0.708 |
| 75 | Yes | 0.712 | 0.409 | 0.675 |

**Table S3B. Observed and parallel-analysis eigenvalues**

| Component | Observed eigenvalue | 95th-percentile random eigenvalue | Decision |
| --- | --- | --- | --- |
| 1 | 11.101 | 1.508 | Retain |
| 2 | 1.182 | 1.404 | Do not retain |
| 3 | 0.703 | 1.328 | Do not retain |
| 4 | 0.592 | 1.268 | Do not retain |
| 5 | 0.549 | 1.217 | Do not retain |
| 6 | 0.452 | 1.164 | Do not retain |
| 7 | 0.414 | 1.120 | Do not retain |
| 8 | 0.381 | 1.077 | Do not retain |

*Note.* Parallel analysis used 1,000 normally distributed random datasets with n=331 and 18 variables; it retained one dominant component. The first two observed eigenvalues accounted for 68.24% of variance.

**Supplementary Appendix 2. Measurement-Model and Alternative-Scoring Results**

**Table S4. Confirmatory factor analysis fit indices**

| Model | df | Chi-square | Chi-square/df | CFI | TLI | RMSEA | SRMR |
| --- | --- | --- | --- | --- | --- | --- | --- |
| HIA10_two_factor | 34 | 716.35 | 21.07 | 0.967 | 0.956 | 0.080 | 0.033 |
| HIA18_two_factor | 134 | 2449.01 | 18.28 | 0.947 | 0.940 | 0.074 | 0.036 |
| DHL9_two_factor | 26 | 339.61 | 13.06 | 0.983 | 0.976 | 0.062 | 0.023 |
| HRP_two_factor | 19 | 1057.03 | 55.63 | 0.931 | 0.899 | 0.132 | 0.063 |
| ESE_two_factor | 34 | 591.89 | 17.41 | 0.976 | 0.968 | 0.072 | 0.026 |

*Note.* Models used maximum-likelihood estimation based on Pearson correlation matrices. Five-category items were treated as approximately continuous.

**Table S5. Standardized loadings and factor-level reliability evidence**

| Model | Item | Factor | Loading | CR | AVE | Factor correlation |
| --- | --- | --- | --- | --- | --- | --- |
| HIA10_two_factor | 58 | 1 | 0.649 | 0.899 | 0.599 | 0.908 |
| HIA10_two_factor | 60 | 1 | 0.760 |  |  |  |
| HIA10_two_factor | 61 | 1 | 0.793 |  |  |  |
| HIA10_two_factor | 63 | 1 | 0.802 |  |  |  |
| HIA10_two_factor | 64 | 1 | 0.836 |  |  |  |
| HIA10_two_factor | 67 | 1 | 0.789 |  |  |  |
| HIA10_two_factor | 68 | 2 | 0.784 | 0.877 | 0.641 |  |
| HIA10_two_factor | 71 | 2 | 0.809 |  |  |  |
| HIA10_two_factor | 72 | 2 | 0.821 |  |  |  |
| HIA10_two_factor | 75 | 2 | 0.787 |  |  |  |
| DHL9_two_factor | 89 | 1 | 0.737 | 0.900 | 0.644 | 0.828 |
| DHL9_two_factor | 90 | 1 | 0.802 |  |  |  |
| DHL9_two_factor | 91 | 1 | 0.834 |  |  |  |
| DHL9_two_factor | 92 | 1 | 0.822 |  |  |  |
| DHL9_two_factor | 93 | 1 | 0.814 |  |  |  |
| DHL9_two_factor | 97 | 2 | 0.765 | 0.877 | 0.640 |  |
| DHL9_two_factor | 98 | 2 | 0.803 |  |  |  |
| DHL9_two_factor | 99 | 2 | 0.829 |  |  |  |
| DHL9_two_factor | 100 | 2 | 0.803 |  |  |  |
| HRP_two_factor | 102 | 1 | 0.609 | 0.809 | 0.517 | 0.876 |
| HRP_two_factor | 103 | 1 | 0.671 |  |  |  |
| HRP_two_factor | 104 | 1 | 0.779 |  |  |  |
| HRP_two_factor | 105 | 1 | 0.801 |  |  |  |
| HRP_two_factor | 106 | 2 | 0.821 | 0.898 | 0.688 |  |
| HRP_two_factor | 107 | 2 | 0.826 |  |  |  |
| HRP_two_factor | 108 | 2 | 0.842 |  |  |  |
| HRP_two_factor | 109 | 2 | 0.828 |  |  |  |
| ESE_two_factor | 110 | 1 | 0.726 | 0.914 | 0.639 | 0.917 |
| ESE_two_factor | 111 | 1 | 0.741 |  |  |  |
| ESE_two_factor | 112 | 1 | 0.825 |  |  |  |
| ESE_two_factor | 113 | 1 | 0.838 |  |  |  |
| ESE_two_factor | 114 | 1 | 0.837 |  |  |  |
| ESE_two_factor | 115 | 1 | 0.820 |  |  |  |
| ESE_two_factor | 116 | 2 | 0.828 | 0.898 | 0.687 |  |
| ESE_two_factor | 117 | 2 | 0.842 |  |  |  |
| ESE_two_factor | 118 | 2 | 0.847 |  |  |  |
| ESE_two_factor | 119 | 2 | 0.797 |  |  |  |

**Table S6. Alternative Digital Health Literacy scoring results**

| Scoring specification | Items | Mean | SD | Observed range | Alpha | r with DHL9 |
| --- | --- | --- | --- | --- | --- | --- |
| DHL9 primary | 9 | 29.215 | 6.253 | 9–45 | 0.925 | 1.000 |
| DHL12 original-direction | 12 | 38.560 | 7.890 | 12–60 | 0.930 | 0.979 |
| DHL12 reverse Q94–Q96 | 12 | 37.870 | 5.050 | 20–60 | 0.697 | 0.947 |

*Note.* The reverse-scored 12-item specification had substantially lower internal consistency. Both 12-item scores were retained only for sensitivity analyses.

**Supplementary Appendix 3. Mediation and Sensitivity Analyses**

**Table S7. Expanded regression coefficients for the adjusted mediation equations**

| Equation | Predictor | B | SE | t | P | 95% CI lower | 95% CI upper |
| --- | --- | --- | --- | --- | --- | --- | --- |
| HRP | Intercept | 9.4657 | 0.6421 | 14.741 | <.001 | 8.2067 | 10.7247 |
| HRP | Internet-use days | 0.3200 | 0.0587 | 5.449 | <.001 | 0.2049 | 0.4352 |
| HRP | Male | -0.6618 | 0.1985 | -3.335 | <.001 | -1.0509 | -0.2727 |
| HRP | Junior | 0.2275 | 0.2449 | 0.929 | .353 | -0.2527 | 0.7077 |
| HRP | Sophomore | 0.1974 | 0.2216 | 0.891 | .373 | -0.2371 | 0.6319 |
| HRP | Senior | 0.3805 | 0.2991 | 1.272 | .203 | -0.2060 | 0.9671 |
| HRP | Humanities | -0.2734 | 0.2433 | -1.124 | .261 | -0.7504 | 0.2036 |
| HRP | Science | -0.7268 | 0.2705 | -2.687 | .007 | -1.2571 | -0.1965 |
| HRP | Arts & sports | -0.6638 | 0.2597 | -2.556 | .011 | -1.1731 | -0.1545 |
| HRP | Rural | 0.3567 | 0.2425 | 1.471 | .141 | -0.1188 | 0.8321 |
| HRP | Urban | 0.5587 | 0.2513 | 2.223 | .026 | 0.0660 | 1.0514 |
| HRP | Economic difficulty | 0.3031 | 0.1896 | 1.599 | .110 | -0.0686 | 0.6748 |
| HRP | Economically affluent | 0.4472 | 0.7068 | 0.633 | .527 | -0.9387 | 1.8330 |
| HRP | DHL9 | 0.5110 | 0.0140 | 36.498 | <.001 | 0.4835 | 0.5384 |
| ESE | Intercept | 13.6627 | 1.0017 | 13.639 | <.001 | 11.6985 | 15.6268 |
| ESE | Internet-use days | -0.1719 | 0.0890 | -1.932 | .054 | -0.3465 | 0.0026 |
| ESE | Male | 1.6469 | 0.2999 | 5.491 | <.001 | 1.0588 | 2.2349 |
| ESE | Junior | -0.4676 | 0.3695 | -1.265 | .206 | -1.1922 | 0.2570 |
| ESE | Sophomore | -0.3575 | 0.3344 | -1.069 | .285 | -1.0131 | 0.2981 |
| ESE | Senior | -0.5904 | 0.4514 | -1.308 | .191 | -1.4754 | 0.2947 |
| ESE | Humanities | -0.5527 | 0.3671 | -1.505 | .132 | -1.2725 | 0.1671 |
| ESE | Science | -0.7845 | 0.4085 | -1.920 | .055 | -1.5855 | 0.0165 |
| ESE | Arts & sports | 1.7216 | 0.3923 | 4.389 | <.001 | 0.9524 | 2.4907 |
| ESE | Rural | 0.4168 | 0.3659 | 1.139 | .255 | -0.3006 | 1.1343 |
| ESE | Urban | -0.1369 | 0.3794 | -0.361 | .718 | -0.8808 | 0.6071 |
| ESE | Economic difficulty | -0.3226 | 0.2861 | -1.128 | .260 | -0.8836 | 0.2384 |
| ESE | Economically affluent | -0.2068 | 1.0664 | -0.194 | .846 | -2.2977 | 1.8841 |
| ESE | DHL9 | 0.4535 | 0.0252 | 17.985 | <.001 | 0.4040 | 0.5029 |
| ESE | HRP | 0.2268 | 0.0269 | 8.417 | <.001 | 0.1740 | 0.2797 |
| HIA10 | Intercept | 9.9057 | 1.0338 | 9.582 | <.001 | 7.8787 | 11.9328 |
| HIA10 | Internet-use days | 0.0280 | 0.0893 | 0.314 | .754 | -0.1471 | 0.2031 |
| HIA10 | Male | -0.2905 | 0.3022 | -0.961 | .336 | -0.8830 | 0.3020 |
| HIA10 | Junior | -0.0175 | 0.3706 | -0.047 | .962 | -0.7442 | 0.7092 |
| HIA10 | Sophomore | 0.4503 | 0.3353 | 1.343 | .179 | -0.2071 | 1.1078 |
| HIA10 | Senior | -0.1821 | 0.4527 | -0.402 | .688 | -1.0698 | 0.7056 |
| HIA10 | Humanities | 0.8189 | 0.3682 | 2.224 | .026 | 0.0969 | 1.5409 |
| HIA10 | Science | 0.4709 | 0.4099 | 1.149 | .251 | -0.3327 | 1.2746 |
| HIA10 | Arts & sports | 0.1159 | 0.3945 | 0.294 | .769 | -0.6576 | 0.8895 |
| HIA10 | Rural | -0.2164 | 0.3670 | -0.590 | .556 | -0.9359 | 0.5032 |
| HIA10 | Urban | 0.7415 | 0.3804 | 1.949 | .051 | -0.0044 | 1.4875 |
| HIA10 | Economic difficulty | -0.5953 | 0.2869 | -2.075 | .038 | -1.1580 | -0.0327 |
| HIA10 | Economically affluent | 1.0245 | 1.0693 | 0.958 | .338 | -1.0720 | 3.1210 |
| HIA10 | DHL9 | 0.3438 | 0.0266 | 12.947 | <.001 | 0.2917 | 0.3959 |
| HIA10 | HRP | 0.1767 | 0.0273 | 6.467 | <.001 | 0.1231 | 0.2303 |
| HIA10 | ESE | 0.1760 | 0.0179 | 9.826 | <.001 | 0.1409 | 0.2111 |

*Note.* Reference categories were female, freshman, engineering major, township residence, and moderate family economic status. Internet-use days was continuous.

**Table S8. Bootstrap indirect associations across primary model specifications**

| Model | Indirect association | Effect | Bootstrap SE | 95% CI lower | 95% CI upper |
| --- | --- | --- | --- | --- | --- |
| Unadjusted | DHL→HRP→HIA | 0.0971 | 0.0166 | 0.0642 | 0.1292 |
| Unadjusted | DHL→ESE→HIA | 0.0719 | 0.0100 | 0.0527 | 0.0918 |
| Unadjusted | DHL→HRP→ESE→HIA | 0.0179 | 0.0041 | 0.0103 | 0.0264 |
| Unadjusted | Total indirect | 0.1869 | 0.0200 | 0.1466 | 0.2258 |
| Six covariates | DHL→HRP→HIA | 0.0903 | 0.0162 | 0.0581 | 0.1216 |
| Six covariates | DHL→ESE→HIA | 0.0798 | 0.0105 | 0.0594 | 0.1008 |
| Six covariates | DHL→HRP→ESE→HIA | 0.0204 | 0.0043 | 0.0124 | 0.0294 |
| Six covariates | Total indirect | 0.1905 | 0.0202 | 0.1499 | 0.2301 |
| Six covariates + batch | DHL→HRP→HIA | 0.0906 | 0.0163 | 0.0581 | 0.1221 |
| Six covariates + batch | DHL→ESE→HIA | 0.0799 | 0.0105 | 0.0595 | 0.1007 |
| Six covariates + batch | DHL→HRP→ESE→HIA | 0.0204 | 0.0043 | 0.0123 | 0.0293 |
| Six covariates + batch | Total indirect | 0.1909 | 0.0202 | 0.1500 | 0.2303 |

*Note.* Percentile bootstrap confidence intervals were based on 5,000 resamples with seed 20260808.

**Table S9. Full-scale and alternative-scoring sensitivity models**

| Model | Total | Direct | Ind1 | Ind2 | Ind3 | Total indirect | Outcome R-squared |
| --- | --- | --- | --- | --- | --- | --- | --- |
| primary_unadjusted | 0.5473 | 0.3604 | 0.0971 | 0.0719 | 0.0179 | 0.1869 | 0.211 |
| primary_six_covariates | 0.5343 | 0.3438 | 0.0903 | 0.0798 | 0.0204 | 0.1905 | 0.221 |
| primary_plus_batch | 0.5346 | 0.3437 | 0.0906 | 0.0799 | 0.0204 | 0.1909 | 0.221 |
| sensitivity_HIA18_DHL9 | 0.9271 | 0.5980 | 0.1407 | 0.1501 | 0.0384 | 0.3291 | 0.213 |
| sensitivity_HIA10_DHL12_original | 0.4179 | 0.2638 | 0.0734 | 0.0647 | 0.0159 | 0.1540 | 0.218 |
| sensitivity_HIA10_DHL12_reversed | 0.6146 | 0.3729 | 0.1206 | 0.0890 | 0.0320 | 0.2417 | 0.215 |

*Note.* Ind1=DHL→HRP→HIA; Ind2=DHL→ESE→HIA; Ind3=DHL→HRP→ESE→HIA. Sensitivity-model indirect confidence intervals were not used for primary inference; point estimates are shown to evaluate robustness of magnitude and direction.

**Supplementary Appendix 4. Expanded Descriptive and Group-Comparison Results**

**Table S10. Complete demographic group means and omnibus comparisons**

| Outcome | Characteristic | Group | n | Mean | SD | Method | Statistic | P | Eta squared |
| --- | --- | --- | --- | --- | --- | --- | --- | --- | --- |
| Health information adoption | Academic year | Freshman | 837 | 30.951 | 7.997 | One-way ANOVA | 1.498 | .213 | .001 |
| Health information adoption | Academic year | Junior | 731 | 30.598 | 8.322 |  |  |  |  |
| Health information adoption | Academic year | Sophomore | 1174 | 31.100 | 8.305 |  |  |  |  |
| Health information adoption | Academic year | Senior | 406 | 30.187 | 8.348 |  |  |  |  |
| Health information adoption | Major category | Engineering | 1007 | 30.260 | 8.358 | One-way ANOVA | 6.867 | <.001 | .007 |
| Health information adoption | Major category | Humanities | 1007 | 31.784 | 7.849 |  |  |  |  |
| Health information adoption | Major category | Science | 521 | 30.560 | 8.223 |  |  |  |  |
| Health information adoption | Major category | Arts and sports | 613 | 30.408 | 8.542 |  |  |  |  |
| Health information adoption | Residential background | Township | 560 | 30.725 | 8.148 | One-way ANOVA | 10.454 | <.001 | .007 |
| Health information adoption | Residential background | Rural | 1477 | 30.209 | 7.934 |  |  |  |  |
| Health information adoption | Residential background | Urban | 1111 | 31.697 | 8.598 |  |  |  |  |
| Health information adoption | Family economic status | Moderate | 2017 | 31.322 | 8.270 | Welch ANOVA | 12.618 | <.001 | .008 |
| Health information adoption | Family economic status | Low | 1083 | 29.828 | 7.959 |  |  |  |  |
| Health information adoption | Family economic status | High | 48 | 32.479 | 10.677 |  |  |  |  |
| Digital health literacy | Academic year | Freshman | 837 | 29.644 | 6.114 | One-way ANOVA | 2.242 | .081 | .002 |
| Digital health literacy | Academic year | Junior | 731 | 29.170 | 6.314 |  |  |  |  |
| Digital health literacy | Academic year | Sophomore | 1174 | 29.101 | 6.291 |  |  |  |  |
| Digital health literacy | Academic year | Senior | 406 | 28.739 | 6.291 |  |  |  |  |
| Digital health literacy | Major category | Engineering | 1007 | 28.922 | 6.120 | Welch ANOVA | 13.045 | <.001 | .012 |
| Digital health literacy | Major category | Humanities | 1007 | 30.138 | 6.206 |  |  |  |  |
| Digital health literacy | Major category | Science | 521 | 29.144 | 6.284 |  |  |  |  |
| Digital health literacy | Major category | Arts and sports | 613 | 28.240 | 6.337 |  |  |  |  |
| Digital health literacy | Residential background | Township | 560 | 29.387 | 5.777 | One-way ANOVA | 2.538 | .079 | .002 |
| Digital health literacy | Residential background | Rural | 1477 | 28.950 | 6.084 |  |  |  |  |
| Digital health literacy | Residential background | Urban | 1111 | 29.480 | 6.683 |  |  |  |  |
| Digital health literacy | Family economic status | Moderate | 2017 | 29.614 | 6.192 | Welch ANOVA | 12.092 | <.001 | .008 |
| Digital health literacy | Family economic status | Low | 1083 | 28.464 | 6.197 |  |  |  |  |
| Digital health literacy | Family economic status | High | 48 | 29.396 | 8.358 |  |  |  |  |
| Health risk perception | Academic year | Freshman | 837 | 26.491 | 5.784 | One-way ANOVA | 0.315 | .814 | .000 |
| Health risk perception | Academic year | Junior | 731 | 26.502 | 5.887 |  |  |  |  |
| Health risk perception | Academic year | Sophomore | 1174 | 26.415 | 5.897 |  |  |  |  |
| Health risk perception | Academic year | Senior | 406 | 26.180 | 5.882 |  |  |  |  |
| Health risk perception | Major category | Engineering | 1007 | 26.393 | 5.746 | One-way ANOVA | 9.406 | <.001 | .009 |
| Health risk perception | Major category | Humanities | 1007 | 27.137 | 5.661 |  |  |  |  |
| Health risk perception | Major category | Science | 521 | 26.015 | 6.108 |  |  |  |  |
| Health risk perception | Major category | Arts and sports | 613 | 25.656 | 6.036 |  |  |  |  |
| Health risk perception | Residential background | Township | 560 | 26.180 | 5.552 | Welch ANOVA | 2.823 | .060 | .002 |
| Health risk perception | Residential background | Rural | 1477 | 26.263 | 5.773 |  |  |  |  |
| Health risk perception | Residential background | Urban | 1111 | 26.764 | 6.113 |  |  |  |  |
| Health risk perception | Family economic status | Moderate | 2017 | 26.594 | 5.647 | One-way ANOVA | 2.695 | .068 | .002 |
| Health risk perception | Family economic status | Low | 1083 | 26.092 | 6.168 |  |  |  |  |
| Health risk perception | Family economic status | High | 48 | 26.812 | 7.254 |  |  |  |  |
| Exercise self-efficacy | Academic year | Freshman | 837 | 32.792 | 8.013 | One-way ANOVA | 1.308 | .270 | .001 |
| Exercise self-efficacy | Academic year | Junior | 731 | 32.038 | 8.179 |  |  |  |  |
| Exercise self-efficacy | Academic year | Sophomore | 1174 | 32.315 | 8.100 |  |  |  |  |
| Exercise self-efficacy | Academic year | Senior | 406 | 32.099 | 8.644 |  |  |  |  |
| Exercise self-efficacy | Major category | Engineering | 1007 | 32.715 | 7.780 | Welch ANOVA | 6.431 | <.001 | .007 |
| Exercise self-efficacy | Major category | Humanities | 1007 | 31.886 | 7.939 |  |  |  |  |
| Exercise self-efficacy | Major category | Science | 521 | 31.415 | 8.582 |  |  |  |  |
| Exercise self-efficacy | Major category | Arts and sports | 613 | 33.307 | 8.671 |  |  |  |  |
| Exercise self-efficacy | Residential background | Township | 560 | 32.093 | 7.494 | Welch ANOVA | 0.518 | .596 | .000 |
| Exercise self-efficacy | Residential background | Rural | 1477 | 32.474 | 7.949 |  |  |  |  |
| Exercise self-efficacy | Residential background | Urban | 1111 | 32.314 | 8.764 |  |  |  |  |
| Exercise self-efficacy | Family economic status | Moderate | 2017 | 32.540 | 8.124 | One-way ANOVA | 1.548 | .213 | .001 |
| Exercise self-efficacy | Family economic status | Low | 1083 | 32.000 | 8.141 |  |  |  |  |
| Exercise self-efficacy | Family economic status | High | 48 | 32.229 | 10.317 |  |  |  |  |

*Note.* Welch ANOVA was used when homogeneity was violated; otherwise conventional one-way ANOVA was used.

**Table S11. Post-hoc comparisons for statistically detectable omnibus tests**

| Outcome | Characteristic | Group 1 | Group 2 | Mean difference | Method | Adjusted P |
| --- | --- | --- | --- | --- | --- | --- |
| Health information adoption | Major category | Engineering | Humanities | -1.523 | Tukey HSD | <.001 |
| Health information adoption | Major category | Engineering | Science | -0.300 | Tukey HSD | .906 |
| Health information adoption | Major category | Engineering | Arts and sports | -0.148 | Tukey HSD | .985 |
| Health information adoption | Major category | Humanities | Science | 1.223 | Tukey HSD | .030 |
| Health information adoption | Major category | Humanities | Arts and sports | 1.376 | Tukey HSD | .006 |
| Health information adoption | Major category | Science | Arts and sports | 0.153 | Tukey HSD | .989 |
| Health information adoption | Residential background | Township | Rural | 0.516 | Tukey HSD | .415 |
| Health information adoption | Residential background | Township | Urban | -0.972 | Tukey HSD | .058 |
| Health information adoption | Residential background | Rural | Urban | -1.487 | Tukey HSD | <.001 |
| Health information adoption | Family economic status | Moderate | Low | 1.494 | Games-Howell | <.001 |
| Health information adoption | Family economic status | Moderate | High | -1.157 | Games-Howell | .738 |
| Health information adoption | Family economic status | Low | High | -2.651 | Games-Howell | .216 |
| Digital health literacy | Major category | Engineering | Humanities | -1.216 | Games-Howell | <.001 |
| Digital health literacy | Major category | Engineering | Science | -0.222 | Games-Howell | .911 |
| Digital health literacy | Major category | Engineering | Arts and sports | 0.682 | Games-Howell | .145 |
| Digital health literacy | Major category | Humanities | Science | 0.994 | Games-Howell | .017 |
| Digital health literacy | Major category | Humanities | Arts and sports | 1.898 | Games-Howell | <.001 |
| Digital health literacy | Major category | Science | Arts and sports | 0.904 | Games-Howell | .077 |
| Digital health literacy | Family economic status | Moderate | Low | 1.150 | Games-Howell | <.001 |
| Digital health literacy | Family economic status | Moderate | High | 0.218 | Games-Howell | .982 |
| Digital health literacy | Family economic status | Low | High | -0.932 | Games-Howell | .727 |
| Digital health literacy | Survey batch | Formal survey batch 1 | Formal survey batch 2 | -0.818 | Games-Howell | .018 |
| Digital health literacy | Survey batch | Formal survey batch 1 | Formal survey batch 3 | -1.006 | Games-Howell | <.001 |
| Digital health literacy | Survey batch | Formal survey batch 2 | Formal survey batch 3 | -0.188 | Games-Howell | .771 |
| Health risk perception | Major category | Engineering | Humanities | -0.744 | Tukey HSD | .022 |
| Health risk perception | Major category | Engineering | Science | 0.378 | Tukey HSD | .627 |
| Health risk perception | Major category | Engineering | Arts and sports | 0.737 | Tukey HSD | .066 |
| Health risk perception | Major category | Humanities | Science | 1.122 | Tukey HSD | .002 |
| Health risk perception | Major category | Humanities | Arts and sports | 1.481 | Tukey HSD | <.001 |
| Health risk perception | Major category | Science | Arts and sports | 0.360 | Tukey HSD | .730 |
| Health risk perception | Survey batch | Formal survey batch 1 | Formal survey batch 2 | -0.273 | Tukey HSD | .595 |
| Health risk perception | Survey batch | Formal survey batch 1 | Formal survey batch 3 | -0.988 | Tukey HSD | <.001 |
| Health risk perception | Survey batch | Formal survey batch 2 | Formal survey batch 3 | -0.714 | Tukey HSD | .014 |
| Exercise self-efficacy | Major category | Engineering | Humanities | 0.829 | Games-Howell | .084 |
| Exercise self-efficacy | Major category | Engineering | Science | 1.300 | Games-Howell | .020 |
| Exercise self-efficacy | Major category | Engineering | Arts and sports | -0.592 | Games-Howell | .510 |
| Exercise self-efficacy | Major category | Humanities | Science | 0.471 | Games-Howell | .724 |
| Exercise self-efficacy | Major category | Humanities | Arts and sports | -1.421 | Games-Howell | .005 |
| Exercise self-efficacy | Major category | Science | Arts and sports | -1.892 | Games-Howell | .001 |

**Table S12. Survey-batch comparisons for the primary variables**

| Outcome | Batch | n | Mean | SD | Method | Statistic | P | Eta squared |
| --- | --- | --- | --- | --- | --- | --- | --- | --- |
| Health information adoption | Formal survey batch 1 | 897 | 30.563 | 8.493 | One-way ANOVA | 0.659 | .517 | .000 |
| Health information adoption | Formal survey batch 2 | 836 | 30.976 | 8.430 |  |  |  |  |
| Health information adoption | Formal survey batch 3 | 1415 | 30.904 | 7.952 |  |  |  |  |
| Digital health literacy | Formal survey batch 1 | 897 | 28.545 | 6.233 | Welch ANOVA | 7.478 | <.001 | .005 |
| Digital health literacy | Formal survey batch 2 | 836 | 29.364 | 6.279 |  |  |  |  |
| Digital health literacy | Formal survey batch 3 | 1415 | 29.551 | 6.223 |  |  |  |  |
| Health risk perception | Formal survey batch 1 | 897 | 25.909 | 5.816 | One-way ANOVA | 8.816 | <.001 | .006 |
| Health risk perception | Formal survey batch 2 | 836 | 26.182 | 5.979 |  |  |  |  |
| Health risk perception | Formal survey batch 3 | 1415 | 26.896 | 5.786 |  |  |  |  |
| Exercise self-efficacy | Formal survey batch 1 | 897 | 32.477 | 8.478 | One-way ANOVA | 0.169 | .845 | .000 |
| Exercise self-efficacy | Formal survey batch 2 | 836 | 32.340 | 8.335 |  |  |  |  |
| Exercise self-efficacy | Formal survey batch 3 | 1415 | 32.275 | 7.869 |  |  |  |  |

*Note.* Batch differences were small. Batch was included only in a sensitivity mediation model.
